# Global and regional impact of health determinants on life expectancy and health-adjusted life expectancy, 2000–2018: an econometric analysis based on the Global Burden of Disease study 2019

**DOI:** 10.3389/fpubh.2025.1566469

**Published:** 2025-04-17

**Authors:** Kamran Irandoust, Rajabali Daroudi, Maryam Tajvar, Mehdi Yaseri

**Affiliations:** ^1^Department of Health Management, Policy, and Economics, School of Public Health, Tehran University of Medical Sciences, Tehran, Iran; ^2^Department of Epidemiology and Biostatistics, School of Public Health, Tehran University of Medical Sciences, Tehran, Iran

**Keywords:** determinants of health, social determinants of health (SDOH), life expectancy (LE), health-adjusted life expectancy (HALE), Global Burden of Disease (GBD)

## Abstract

**Background:**

The health status of a population is influenced by multiple determinants, including clinical care, health behaviors, the physical environment, and socioeconomic factors. This study examines the impact of these determinants on life expectancy (LE) and health-adjusted life expectancy (HALE) at both regional and global levels using econometric analysis.

**Methods:**

This ecological study included all 194 WHO member countries from 2000 to 2018. The County Health Rankings Model was used to identify key health determinants. Thirty-six indicators were selected to measure these determinants, with data collected from the World Bank, World Health Observatory, Global Health Expenditure Database, Gapminder, United Nations Human Development Reports, and Global Burden of Disease Studies. LE and HALE were used as health status indicators, with data extracted from the Global Burden of Disease Study 2019 database. A multilevel mixed-effects linear regression model was applied for statistical analysis using Stata 16 software.

**Results:**

At the global level, the regression coefficients (β) with LE and HALE were 0.09 and 0.10 for education, −0.04 and −0.10 for injuries, 0.5 and 0.6 for urbanization, 0.10 and 0.8 for access to basic drinking water, −0.5 and −0.4 for drug use, 0.4 and 0.3 for obesity, and −0.15 and −0.16 for sexually transmitted infections, respectively. Sexually transmitted infections (β = −0.25) in the African region, access to basic drinking water (β = 0.30), alcohol consumption (β = −0.06), and drug use (β = −0.02) in the Americas, injuries (β = −0.16), air pollution (β = −0.10), and obesity (β = −0.24) in the Eastern Mediterranean, urbanization (β = 0.08) in Southeast Asia, and education (β = 0.36) and smoking (β = −0.06) in the Western Pacific had the greatest impact on HALE compared to other regions (*P* < 0.05).

**Conclusion:**

To reduce inequalities, improve public health outcomes, and ensure efficient resource allocation, global and interregional policies should prioritize the determinants with the highest β values for health indicators in each region. These determinants are expected to yield greater marginal health benefits, making investments in them more cost-effective.

## 1 Introduction

Concepts of health have developed at different stages throughout the history of public health development. In today's world, health is not a unique trait determined by genetic makeup alone. Rather, health is mainly shaped by a set of environmental and social factors that enable the unique expression of the genetic structure (1). Based on socio-ecological thinking, human health and its determinants are related and different determinants of health lead to different patterns in the burden of diseases (2, 3). In fact, the health status of a population can be determined by a combination of health determinants including health care, lifestyle, physical environment and socioeconomic factors (4, 32). These preventable factors influence health opportunities, health-seeking behaviors and lifestyles, as well as disease outcomes (5), and are considered one of the most effective public health strategies to reduce disease and health harms (6).

Advances in public health and rapid advances in socio-economic and medical technologies over the past 30 years have resulted in gains in life and continued improvement in global life expectancy (LE). However, long survival does not equal good quality of life (7, 8). Therefore, healthy life expectancy or health-adjusted life expectancy (HALE) has provided a new perspective to assess quality of life (9). HALE aims to summarize the number of years that a person can expect to live in full health by taking into account years lived in less than full health due to disease and/or injury (4). The World Health Organization (WHO) reported an increase of 6.6 and 5.4 years from 2000 to 2019 in LE and HALE at birth, indicating that longevity and healthy LE may differ (10). However, the correlation between HALE and raw LE indicators is very high and significant (4). LE and HALE have increased in many parts of the world (11). However, these gains in life years and years lived in good health are not equally distributed among all population groups, and there are inequalities according to factors such as socioeconomic status (12). Reports on health equity have shown that differences in health are due to social differences. According to these reports, low social classes have higher mortality rates and more chronic diseases than high social classes (13, 14). The Commission on Social Determinants of Health of the WHO, in one of its final reports on closing the gap and inequality in one generation, concluded that achieving health equity requires action on the conditions in which people are born, grow, work, live, and age. It also confirmed the structural drivers of these conditions at the global, regional, national and local levels (15).

Since understanding the distribution and changes of these inequalities over time is increasingly important for developing policies aimed at advancing health equity and health promotion, as well as prioritizing efficient and effective resource allocation, this study investigates the impact of health determinants on LE and HALE at the global and regional levels. The study is framed within the conceptual framework of socio-ecological models, which suggest that human health is influenced by a wide range of factors across different levels. Using econometric analysis of data from 194 WHO member countries between 2000 and 2018, the study identifies the health determinants that have the greatest impact on health in each region.

## 2 Methods

The present ecological study is a retrospective descriptive-analytical investigation conducted using data from 2000 to 2018. The study encompassed all 194 WHO Member States as its statistical population. Inclusion in the study was based on a country's WHO membership, while exclusion was determined by non-membership in the WHO. To explore the influence of global and regional determinants on LE and HALE, this research followed an eight- stage process.

**Stage 1—Selecting the health determinants model**: first, to select the determinants of health, it was necessary to identify them according to a specific framework or model. In this study, considering the nature and requirements of the research, we examined and compared various models and ultimately chose the County Health Rankings & Roadmaps Model to guide this study (see Supplementary Figure 1). According to this model, 13 health determinants can be identified, categorized into four general groups: clinical care, social and economic factors, physical environment, and health behaviors (16, 32).

**Stage 2—Selection of indicators according to the model**: at this stage, based on the model selected in Stage 1, we explored and identified indicators to measure the variables of the model. To this end, by reviewing similar studies and global databases, 36 indicators were chosen to measure the 13 health determinants, and 2 indicators of LE and HALE were selected to measure health status, as detailed in Table 1.

**Table 1:** Selected indicators to measure health outcomes and health determinants.

**Dimension**	**Component**	**Indicators**	**Description**	**DA %**
Health outcomes	Length of life	LE	Years, total	100.00
	Quality of life	HALE	Years, all age	99.48
Clinical care	Access to care	Current health expenditure	Per capita, PPP (current international $)	90.31
	Quality to care	Domestic general government health exp	Per capita, PPP (current international $)	90.80
Social and economic factors	Income	Gross domestic product (GDP)	Per capita, PPP (constant 2017 international $)	93.24
		Income index^**^	Income index-HDI	97.21
		Income inequality^*^	GINI index (world bank estimate)	33.97
	Education	Years of schooling^**^	Mean years of schooling (years)	94.38
		Literacy^*^	Rate, adult total (% of people ages 15 and above)	18.50
		Education index	Education index-HDI	94.00
	Employment	Unemployment	% of total labor force (modeled ILO estimate)	82.09
	Community safety	Injury prevalence	Rate, Age Standardized	100.00
		Intentional homicides^*^	Intentional homicides (per 100,000 people)	62.56
	Family and social support	Social protection^*^	CPIA social protection rating (1 = low to 6 = high)	28.51
		Coverage of social safety net program^*^	% population–coverage (%)–all social assistance	10.15
Physical environment	Housing and transit	Urban population (urbanization)	Urban population (% of total population)	98.78
		Population density^**^	Population density (people per sq. km of land area)	97.75
		Poverty^*^	Poverty gap at $3.20 a day (2011 PPP) (%)	34.16
	Air and water quality	PM2.5 air pollution^*^	Mean annual exposure (micrograms per cubic meter)	50.46
		CO_2_ emissions^*^	CO_2_ emissions (metric tons per capita)	74.58
		Air pollution	Summary exposure value per 100, rate, age-standardized	100.00
		Basic drinking-water services	Population using at least (%)	92.11
		Managed drinking water services^*^	People using safely (% of population)	46.85
		Unsafe water, sanitation, hand washing^**^	Summary exposure value per 100, rate, age-standardized	100.00
Health behaviors	Alcohol and drug use	Alcohol consumption^**^	Recorded per capita (15+) (in liters of pure alcohol)	94.93
		Prevalence of Alcohol use disorders^**^	Rate, age-standardized	99.86
		Alcohol use	Summary exposure value per 100, Rate, age-standardized	100.00
		Prevalence of drug use disorders^**^	Rate, age-standardized	99.32
		Drug use	Summary exposure value per 100, Rate, age-standardized	99.92
	Tobacco use	Smoking prevalence^*^	Smoking prevalence, total (ages 15+)	33.45
		Tobacco^**^	Summary exposure value per 100, rate, age-standardized	98.45
		Smoking	Summary exposure value per 100, rate, age-standardized	100.00
	Diet and exercise	Oil consumption^*^	Per capita (tones per year per person)	28.13
		Consumption of iodized salt^*^	Consumption of iodized salt (% of households)	8.11
		Sugar consumption^*^	Sugar per person (g per day)	57.03
		Prevalence of obesity among adults	BMI and greater equal, 30 (age-standardized) (%)	87.17
		Prevalence of overweight^**^	Prevalence of overweight (% of adults)	86.25
	Sexual activity	HIV and sexually transmitted infections	Prevalence, rate, age-standardized	99.48

^*^Indicators where the amount of data availability (DA) was < 80%. ^**^indicators that overlapped.

**Stage 3—Identification and selection of countries by regions**: All WHO member countries, totaling 194 countries, were included in the study. The list of these countries, categorized by region, is provided in Supplementary Table 1.

**Stage 4—Collecting data for selected indicators and countries**: at this stage, data for 36 health determinant indicators were extracted from the databases of the World Bank, World Health Observatory, Global Health Expenditure Database, Gapminder, United Nations Human Development Reports, and Global Burden of Disease Studies. Additionally, data for 2 health status indicators were extracted from the Global Burden of Disease Study 2019 database. It should be noted that for indicators with data available in multiple databases, the database with the most complete data was used. The access links for each of these databases are provided in Supplementary Table 2.

**Stage 5—Creating a panel data file**: in this study, as the data consists of both time-series data (2000–2018) and cross-sectional data (all WHO member countries), the data is categorized as panel data. Excel 2010 software was used to create a panel data file, which includes 36 health determinant indicators and two health status indicators for 194 countries between 2000 and 2018.

**Stage 6—Data refinement**: at this stage, we first checked the accuracy of the data and then removed outlier data. Additionally, we calculated the data availability for each of the indicators, as shown in Table 1. The purpose of removing outlier data was to prevent spurious regressions in the statistical analysis.

**Stage 7—Finalizing the indicators**: to finalize the indicators of the study, we excluded unnecessary indicators through four steps: First, using a multilevel mixed-effects linear regression model, we performed univariate analysis to test the significant relationships between the 36 health determinant indicators and the 2 health status indicators. Since this was a univariate analysis, a separate model was defined for each health determinant, repeated 72 times using Stata software. After performing this test for all variables, we excluded indicators with a *p*-value above 0.1 (see Supplementary Table 3). Second, indicators with < 80% data availability were excluded from the study (marked with ^*^ in Table 1). Third, overlapping indicators were excluded (marked with ^**^ in Table 1). Fourth, using the multilevel mixed-effects linear regression model, we performed multiple analysis for the remaining indicators, entering all health determinant indicators into the model at once. After the multiple analysis, indicators with a *p* < 0.1 were excluded—this process involved removing the indicator with the highest *p*-value above 0.1 first, then rerunning the model, and repeating this process until no indicator with a *p* < 0.1 remained in the final model. After these four steps, 11 indicators were retained to explain LE, and 10 indicators to explain HALE were entered into the final model for statistical analysis (see Supplementary Table 4).

**Stage 8—Selection of statistical model and data analysis**: at this stage, we transformed both the dependent and independent variables by applying natural logarithms to normalize the data and address any skewness. This transformation was essential to ensure the proper distribution of the variables. We then employed a multilevel mixed-effects linear regression model to analyze the relationship between health determinants (independent variables) and health status (dependent variables), accounting for the hierarchical structure of the data (country-level and time-level).

Based on statistical criteria, the nature of our data, and the study's objectives, we selected a random-effects model. This choice was justified by the significant variation across groups, which allowed us to capture unobserved heterogeneity. Additionally, the random-effects model enabled us to retain time-invariant variables, ensuring a comprehensive analysis.

For data analysis, we used STATA 16 software, and R version 4.0.3 was employed for data visualization.

## 3 Results

### 3.1 Global analysis

The analytical findings of the present study at the global level showed that the regression coefficients (β) with LE and HALE were 0.09 and 0.10 for education, −0.04 and −0.10 for injuries, 0.5 and 0.6 for urbanization, 0.10 and 0.8 for drinking water, −0.5 and −0.4 for drugs, 0.4 and 0.3 for obesity, and −0.15 and −0.16 for sexually transmitted infections, respectively. In this way, at the global level, the effect of education, injuries, and urbanization variables on HALE was greater than LE, but the effect of drinking water, drugs, and obesity variables on LE was greater than HALE (*P* < 0.05; Table 2).

**Table 2:** Regression analysis results at the global level (2000–2018).

**Variables**	**Linear regression**			**Log-log linear regression**		
	β	**CI (95%)**	***P*** **-value**	β	**CI (95%)**	***P*** **-value**
**LE**						
Education Index	4.02	2.12 to 5.91	< 0.001	0.09	0.07 to 0.10	< 0.001
Unemployment	−0.02	−0.04 to −0.00	< 0.044	0.001	−0.002 to 0.004	0.435
Injury prevalence	−0.00	−0.00 to −0.00	< 0.001	−0.04	−0.05 to −0.02	< 0.001
Urbanization	0.08	0.06 to 0.10	< 0.001	0.05	0.03 to 0.07	< 0.001
Air pollution	−0.08	−0.11 to −0.06	< 0.001	0.003	−0.006 to 0.01	0.489
Basic drinking-water services	0.08	0.060 to 0.09	< 0.001	0.10	0.09 to 0.12	< 0.001
Alcohol use	−0.28	−0.36 to −0.20	< 0.001	0.003	−0.001 to 0.008	0.102
Drug use	−3.66	−5.44 to −1.87	< 0.001	−0.05	−0.06 to −0.04	< 0.001
Smoking	0.26	0.19 to 0.33	< 0.001	0.03	0.02 to 0.05	< 0.001
Prevalence of obesity	−0.41	−0.46 to −0.35	< 0.001	0.04	0.02 to 0.05	< 0.001
Sexually transmitted infections	−0.00	−0.00 to −0.00	< 0.001	−0.15	−0.17 to −0.12	< 0.001
Year	0.41	0.38 to 0.44	< 0.001	0.002	0.002 to 0.003	< 0.001
**HALE**						
Education Index	5.61	3.91 to 7.31	< 0.001	0.10	0.09 to 0.12	< 0.001
Injury prevalence	−0.00	−0.00 to −0.00	< 0.001	−0.10	−0.11 to −0.08	< 0.001
Urbanization	0.07	0.05 to 0.09	< 0.001	0.06	0.04 to 0.07	< 0.001
Air pollution	−0.09	−0.11 to −0.07	< 0.001	−0.002	−0.01 to 0.01	0.595
Basic drinking-water services	0.05	0.03 to 0.06	< 0.001	0.08	0.06 to 0.09	< 0.001
Alcohol use	−0.14	−0.21 to −0.07	0.007	0.003	−0.001 to 0.008	0.137
Drug use	−4.01	−5.65 to −2.38	< 0.001	−0.05	−0.06 to −0.03	< 0.001
Smoking	0.15	0.08 to 0.21	< 0.001	0.006	−0.01 to 0.02	0.398
Prevalence of obesity	−0.38	−0.43 to −0.34	< 0.001	0.03	0.02 to 0.04	< 0.001
Sexually transmitted infections	−0.00	−0.00 to −0.00	< 0.001	−0.16	−0.18 to −0.14	< 0.001
Year	0.29	0.27 to 0.32	< 0.001	0.001	0.000 to 0.002	< 0.001

As mentioned in the global findings above, the variables of sexually transmitted infections (β = −0.15), drinking water (β = 0.10), and education (β = 0.09) have the greatest role in explaining LE. In this way, it can be stated that a one-percent increase in sexually transmitted infections is expected to decrease LE by 0.15%. Similarly, a one-percent increase in basic drinking water services and education is expected to increase LE by 0.10% and 0.9%, respectively. The highest correlation with HALE was also related to the variables of sexually transmitted infections (β = −0.16), injury prevalence (β = −0.10), and education (β = 0.10) (*P* < 0.05; Table 2).

### 3.2 Regional analysis

The analytical findings of the present study at the regional level showed that the variables of education (β = 0.14), air pollution (β = −0.10), smoking (β = −0.02), and sexually transmitted infections (β = −0.22) in the African region, variables of drinking water (β = 0.30) and alcohol (β = −0.04) in the Americas region, variables of injury (β = −0.17) and obesity (β = −0.20) in the Eastern Mediterranean region, variables of urbanization (β = 0.04) and drugs (β = −0.01) in the European region, and variable of unemployment (β = −0.02) in South-East Asia region had the most significant effects on LE compared to other regions (*P* < 0.05; Table 3).

**Table 3:** Regression analysis results at the regional level (2000–2018).

**Variables**	**WHO regions**					
	**African**	**Americas**	**Eastern Mediterranean**	**Europe**	**South-East Asia**	**Western Pacific**
	β **(*****P*****-value)**	β **(*****P*****-value)**	β **(*****P*****-value)**	β **(*****P*****-value)**	β **(*****P*****-value)**	β **(*****P*****-value)**
**LE**						
Education index	0.14 (< 0.001)	0.09 (< 0.001)	0.10 (< 0.001)	0.10 (< 0.001)	0.11 (< 0.001)	0.12 (< 0.001)
Unemployment	0.005 (0.264)	−0.01 (0.009)	0.001 (0.700)	0.01 (< 0.001)	−0.02 (< 0.001)	0.01 (0.133)
Injury prevalence	0.04 (0.001)	0.02 (0.036)	−0.17 (< 0.001)	−0.04 (< 0.001)	−0.01 (0.112)	0.03 (0.016)
Urbanization	0.02 (0.015)	0.006 (0.399)	−0.04 (< 0.001)	0.04 (< 0.001)	−0.02 (0.028)	−0.05 (< 0.001)
Air pollution	−0.10 (< 0.001)	−0.01 (0.042)	−0.09 (< 0.001)	−0.02 (< 0.001)	−0.08 (< 0.001)	−0.01 (0.263)
Basic drinking-water	0.10 (< 0.001)	0.30 (< 0.001)	0.10 (< 0.001)	0.16 (< 0.001)	−0.03 (0.003)	0.14 (< 0.001)
Alcohol use	−0.02 (< 0.001)	−0.04 (< 0.001)	0.01 (< 0.001)	0.001 (0.700)	0.01 (< 0.001)	−0.00 (0.600)
Drug use	−0.02 (0.113)	−0.004 (0.302)	0.09 (< 0.001)	−0.01 (< 0.001)	0.004 (0.260)	0.02 (0.026)
Smoking	−0.02 (0.001)	0.02 (0.001)	−0.1 (0.154)	0.02 (< 0.001)	−0.1 (0.068)	−0.01 (0.528)
Prevalence of obesity	−0.10 (< 0.001)	−0.02 (0.170)	−0.20 (< 0.001)	−0.06 (< 0.001)	0.04 (< 0.001)	−0.02 (< 0.001)
Sexually transmitted	−0.22 (< 0.001)	−0.04 (0.004)	0.03 (0.005)	−0.16 (< 0.001)	−0.08 (< 0.001)	−0.11 (< 0.001)
Year	0.010 (< 0.001)	0.001 (0.025)	0.013 (< 0.001)	0.003 (< 0.001)	0.000 (0.616)	0.002 (< 0.001)
**HALE**						
Education Index	0.14 (< 0.001)	0.22 (< 0.001)	0.08 (< 0.001)	0.03 (0.008)	0.14 (< 0.001)	0.36 (< 0.001)
Injury prevalence	0.02 (0.054)	−0.00 (0.986)	−0.16 (< 0.001)	−0.03 (< 0.001)	−0.06 (< 0.001)	−0.08 (< 0.001)
Urbanization	0.03 (< 0.001)	0.04 (< 0.001)	−0.02 (0.012)	0.02 (0.004)	0.08 (< 0.001)	−0.09 (< 0.001)
Air pollution	−0.06 (< 0.001)	0.01 (0.176)	−0.10 (< 0.001)	−0.03 (< 0.001)	−0.04 (< 0.001)	−0.02 (< 0.001)
Basic drinking-water	0.08 (< 0.001)	0.30 (< 0.001)	0.13 (< 0.001)	0.09 (< 0.001)	−0.09 (< 0.001)	−0.03 (0.043)
Alcohol use	−0.02 (< 0.001)	−0.06 (< 0.001)	0.02 (< 0.001)	−0.003 (0.284)	−0.02 (< 0.001)	−0.00 (0.815)
Drug use	−0.001 (0.885)	−0.02 (0.005)	0.11 (< 0.001)	−0.01 (< 0.001)	−0.01 (0.053)	0.04 (< 0.001)
Smoking	−0.02 (< 0.001)	0.01 (0.280)	0.06 (< 0.001)	0.04 (< 0.001)	0.07 (< 0.001)	−0.06 (< 0.001)
Prevalence of obesity	−0.11 (< 0.001)	−0.06 (0.001)	−0.24 (< 0.001)	−0.04 (< 0.001)	−0.001 (0.882)	−0.04 (< 0.001)
Sexually transmitted	−0.25 (< 0.001)	−0.04 (0.015)	0.04 (0.002)	−0.14 (< 0.001)	0.002 (845)	−0.18 (< 0.001)
Year	0.009 (< 0.001)	−0.000 (0.971)	0.02 (< 0.001)	0.003 (< 0.001)	0.001 (< 0.001)	−0.000 (0.685)

The analysis based on the HALE also showed that the variables of sexually transmitted infections (β = −0.25) in the African region, drinking water (β = 0.30), alcohol (β = −0.06), and drugs (β = −0.02) in the Americas region, injuries (β = −0.16), air pollution (β = −0.10), and obesity (β = −0.24) in the Eastern Mediterranean region, urbanization (β = 0.08) in the South–East Asia region, and education (β = 0.36) and smoking (β = −0.06) in the Western Pacific region had the most significant effects on HALE compared to other regions (*P* < 0.05; Table 3).

### 3.3 Data visualization

**Figure 1:**
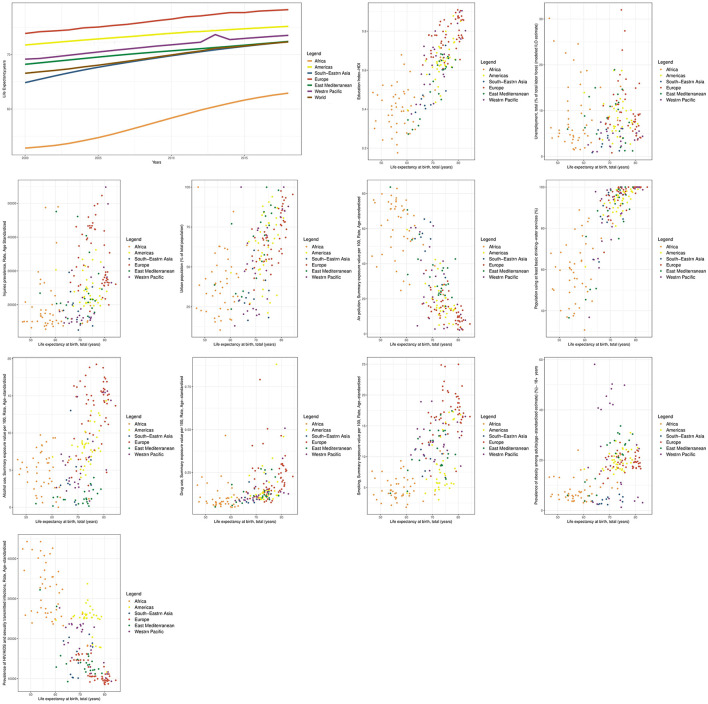
Time trend of LE and scatter plots of its determinants at the global and WHO regional levels.

The graph in Figure 1 shows that the time trend of the LE between 2000 and 2018 maintained a consistent order for all regions and the global average. This order remained constant throughout the period, with the highest values observed in the regions of Europe, the Americas, Western Pacific, Eastern Mediterranean, the global average, South-East Asia, and Africa, respectively (Figure 1).

**Figure 2:**
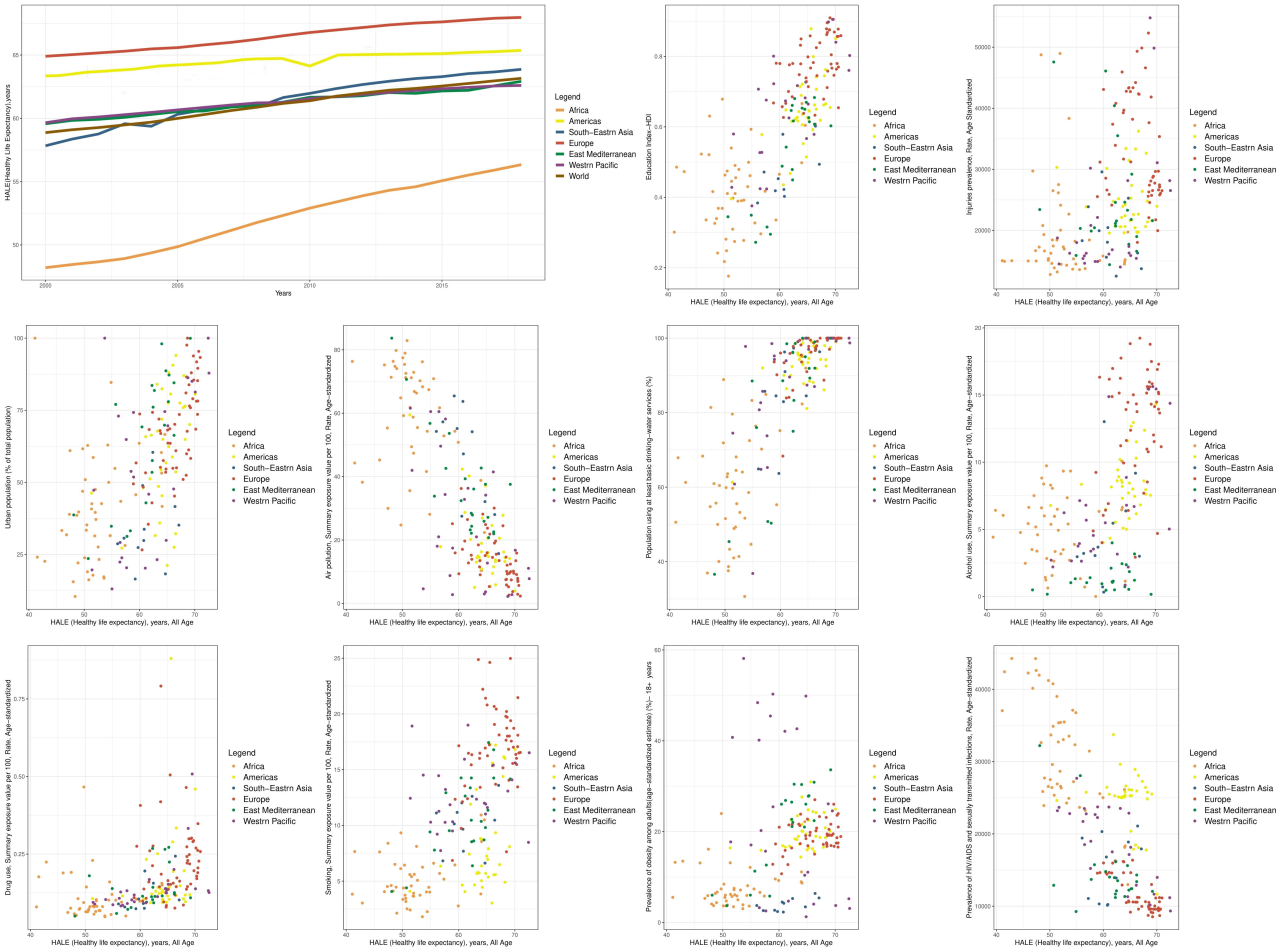
Time trend of HALE and scatter plots of its determinants at the global and WHO regional levels.

Between 2000 and 2018, the HALE trend showed that in 2000, the highest value of the HALE indicator was observed in Europe, the Americas, Western Pacific, Eastern Mediterranean, South-East Asia, and Africa, respectively. By 2018, the highest value of this indicator was in Europe, the Americas, South-East Asia, Eastern Mediterranean, Western Pacific, and Africa, respectively. This indicates an improvement in the position of South-East Asia during this time period (Figure 2).

Comparing the global HALE with regional data, in 2000, Africa and South-East Asia had worse health situations than the global average. In 2018, South-East Asia improved more than the global trend, while the Western Pacific and Eastern Mediterranean regions improved less than the global trend. As a result, Africa, Western Pacific, and Eastern Mediterranean regions had worse situations compared to the global average in 2018 (Figure 2).

The additional graphs in Figures 1, 2 depict the relationship between the LE and HALE indicators with health determinants, categorized by WHO regions. Each data point on the graph represents the average of these indicators for a country from 2000 to 2018. It's worth noting that all the indicators for which scatter diagrams were created, whether through univariate analysis or multiple analysis at the global level, displayed a significant relationship with both the LE and HALE.

## 4 Discussion

The study's global findings revealed that education, injuries, and urbanization had a greater impact on HALE compared to LE, while drinking water, drugs, and obesity had a stronger effect on LE than HALE. This suggests that differences between LE and HALE indicators can partly be explained by the varying influence of health determinants on these indicators (10), Therefore, improving the global status of education, reducing injuries, and enhancing urbanization—given their stronger influence on HALE than on LE—could lead to greater improvements in HALE, thereby reducing the gap between the two indicators. Additionally, the study found that sexually transmitted infections, injury prevalence, and education had the highest correlation with HALE, while sexually transmitted infections, drinking water, and education were most strongly associated with LE. The similarity of some of these results may be due to the high and significant correlation between LE and HALE (4).

In the findings of the present study, a negative relationship was observed between obesity and the LE and HALE indicators in all regions, except in the case of LE in South-East Asia. A possible explanation for this finding is that obesity is one side of the double burden of malnutrition, and in every region except South-East Asia, the number of obese individuals exceeds that of underweight individuals (17). Therefore, the different impact of obesity on LE in South-East Asia may be attributed to the fact that underweight, which is more prevalent in this region, has a greater negative effect on health. As shown in the study by Steensma et al., LE and HALE were significantly lower in the underweight and obesity class 2+ categories than in the normal weight category (18). It is also important to note that due to data limitations, this study used obesity as a proxy indicator instead of diet and exercise. However, considering their role in obesity, it is crucial to recognize that diet and exercise are distinct health behaviors, each independently contributing to morbidity and mortality.

The most significant negative effect of sexually transmitted infections on both LE and HALE was observed in the African region. This heightened impact can be explained by the increased prevalence of unprotected sex, which is often linked to poor socioeconomic conditions and gender-based violence (19). Additionally, education, air pollution, and smoking had the highest correlation with LE in the African region compared to other regions. Therefore, when formulating interregional policies, addressing these variables, particularly sexually transmitted infections, is expected to result in a higher marginal health benefit in the African region compared to others. Several studies have highlighted the impact of lower education on LE (20, 21). In the Western Pacific region, the effect of education and smoking on HALE is more pronounced than in other regions. As a result, these two variables are particularly important in the Western Pacific region, in addition to the African region.

The effect of obesity and injury on both LE and HALE was more significant in the Eastern Mediterranean region compared to other regions. Additionally, the effect of air pollution on HALE was stronger in this region than in others. These findings align with other studies showing that low socioeconomic groups tend to experience higher disability-adjusted life years due to air pollution and high body mass index (22). Furthermore, other studies have shown an increased risk of mortality associated with overweight and obesity (23–25).

The highest β values for drinking water and alcohol variables with HALE and LE, as well as the highest β value for the drug variable with the HALE, were observed in the American region. This suggests that investing in these factors in the American region could yield more cost-effective health outcomes compared to other regions. Studies show that contaminated drinking water transmits various diseases that adversely affect LE, particularly through infant mortality (26, 27). One study also found that alcohol abuse is often associated with smoking and illicit drug use, leading to long-term adverse health outcomes (28).

Additionally, the highest β value for drugs with the LE was found in the European region. Therefore, in addition to the American region, addressing drug use in the European region is also of great importance. In the European region, no variable had a greater impact on the HALE compared to other regions, suggesting that the region has effectively responded to health determinants, possibly due to its economic prosperity, which has resulted in fewer health problems associated with poverty and deprivation compared to other regions (15).

The rapid growth of urbanization and the increase in the number of metropolises have led to numerous environmental and social problems that reduce quality of life and create significant challenges and opportunities for sustainable development in the future (29–31). The findings of the present study showed that the effect of urbanization on HALE differs across regions. It has a negative effect in the Eastern Mediterranean and Western Pacific regions but a positive effect in Africa, the Americas, Europe, and Southeast Asia. Southeast Asia and Europe show the most positive impact on HALE and LE indicators, respectively, from urbanization, while the Western Pacific region has the most negative impact on both indicators.

### 4.1 Limitations

One of the limitations of our study was the incompleteness of data related to certain variables, including family and social support. Consequently, as outlined in the study's methodology, we excluded indicators or variables with < 80% data coverage from the analysis. Additionally, the lack of sufficient data on diet and exercise led to the use of obesity as a proxy indicator, which may overlook the independent effects of these factors. The second limitation arises from the fact that the most recent available data from the Global Burden of Disease (GBD) study at the time of our research was from 2019. As GBD 2021 data had not yet been released at the time of the study, we had to rely on data from 2000 to 2018. This decision is important because, despite significant global changes in recent years, particularly due to the coronavirus disease 2019 (COVID-19) pandemic, these data do not reflect the most recent global health developments. Therefore, the absence of more recent data is a limitation in capturing the full impact of recent events on health determinants and outcomes. The third limitation is associated with the difficulty of comparing various variables within a specific region due to the diverse nature of the indicators. Therefore, in interpreting the findings, we focused on comparing individual variables across regions rather than comparing multiple variables within a single region. The fourth limitation of our study is related to the ecological fallacy inherent in ecological studies, which involves assuming that relationships observed at the overall level apply consistently at the individual level. To mitigate this, we exercised caution when interpreting the results and making inferences at both the global and regional levels, avoiding direct attribution to individual countries or individuals.

## 5 Conclusion

In order to reduce inequalities and gaps in public health, as well as ensure efficient and effective allocation of resources, it is recommended that global and interregional policies pay special attention to the variables or determinants that had the highest β value with health indicators in each region compared to other regions. This is because these determinants probably have a higher marginal health benefit, and investing in them is expected to be more cost-effective.

## Data Availability

The original contributions presented in the study are included in the article/Supplementary material, further inquiries can be directed to the corresponding authors.
