# Supplementary material for: Global and regional impact of health determinants on life expectancy and health-adjusted life expectancy, 2000–2018: an econometric analysis based on the Global Burden of Disease study 2019
Source: Front Public Health. 2025 Apr 17;13:1566469. doi: 10.3389/fpubh.2025.1566469 (PMC12043586; doi:10.3389/fpubh.2025.1566469)
Supplement: Supplementary file 1 [file Data_Sheet_1.docx]

Supplementary Material

# Supplementary Tables

## Supplementary Tables 1 WHO member countries

| **WHO Region** | **194 Member countries** |
| --- | --- |
| **Africa**  **(47 countries)** | Algeria, Angola, Benin, Botswana, Burkina Faso, Burundi, Cabo Verde, Cameroon, Central African Republic, Chad, Comoros, Congo, Cote d'Ivoire, Democratic Republic of Congo, Equatorial Guinea, Eritrea, Eswatini, Ethiopia, Gabon, Gambia, Ghana, Guinea, Guinea-Bissau, Kenya, Lesotho, Liberia, Madagascar, Malawi, Mali, Mauritania, Mauritius, Mozambique, Namibia, Niger, Nigeria, Rwanda, Sao Tome and Principe, Senegal, Seychelles, Sierra Leone, South Africa, South Sudan, Togo, Uganda, United Republic of Tanzania, Zambia, Zimbabwe |
| **Americas**  **(35 countries)** | Antigua and Barbuda, Argentina, Bahamas, Barbados, Belize, Bolivia, Brazil, Canada, Chile, Colombia, Costa Rica, Cuba, Dominica, Dominican Republic, Ecuador, El Salvador, Grenada, Guatemala, Guyana, Haiti, Honduras, Jamaica, Mexico, Nicaragua, Panama, Paraguay, Peru, Saint Kitts and Nevis, Saint Lucia, Saint Vincent and the Grenadines, Suriname, Trinidad and Tobago, United States of America, Uruguay, Venezuela (Bolivarian Republic of) |
| **South-East Asia**  **(11 countries)** | Bangladesh, Bhutan, Democratic People’s Republic of Korea, India, Indonesia, Maldives, Myanmar, Nepal, Sri Lanka, Thailand, Timor-Leste |
| **Europe**  **(53 countries)** | Albania, Andorra, Armenia, Austria, Azerbaijan, Belarus, Belgium, Bosnia and Herzegovina, Bulgaria, Croatia, Cyprus, Czechia, Denmark, Estonia, Finland, France, Georgia, Germany, Greece, Hungary, Iceland, Ireland, Israel, Italy, Kazakhstan, Kyrgyzstan, Latvia, Lithuania, Luxembourg, Malta, Monaco, Montenegro, Netherlands, North Macedonia, Norway, Poland, Portugal, Republic of Moldova, Romania, Russian Federation, San Marino, Serbia, Slovakia, Slovenia, Spain, Sweden, Switzerland, Tajikistan, Turkey, Turkmenistan, Ukraine, United Kingdom of Great Britain and Northern Ireland, Uzbekistan |
| **Eastern Mediterranean**  **(21 countries)** | Afghanistan, Bahrain, Djibouti, Egypt, Iran Islamic Republic of, Iraq, Jordan, Kuwait, Lebanon, Libya, Morocco, Oman, Pakistan, Qatar, Saudi Arabia, Somalia, Sudan, Syrian Arab Republic, Tunisia, United Arab Emirates, Yemen |
| **Western Pacific**  **(27 countries)** | Australia, Brunei Darussalam, Cambodia, China, Cook Islands, Fiji, Japan, Kiribati, Lao Peoples Democratic Republic, Malaysia, Marshall Islands, Micronesia (Federated States of), Mongolia, Nauru, New Zealand, Niue, Palau, Papua New Guinea, Philippines, Republic of Korea, Samoa, Singapore, Solomon Islands, Tonga, Tuvalu, Vanuatu, Viet Nam |

## Supplementary Tables 2 List of databases used in the study and their access link

| **Database** | **Database URL** |
| --- | --- |
| World Bank | <https://databank.worldbank.org/source/world-development-indicators> |
| World Health Observatory | <https://apps.who.int/gho/data/node.imr> |
| Global Health Expenditure Database | <https://apps.who.int/nha/database/Select/Indicators/en> |
| Gapminder | <https://www.gapminder.org/data/> |
| United Nations Human Development Reports | <http://hdr.undp.org/en/data> |
| Global Burden of Disease Studies | <http://ghdx.healthdata.org/gbd-results-tool> |

## Supplementary Tables 3 The results of the univariate test of the multilevel mixed-effects linear regression model

| **Indicators** | | **Coeff** | **CI (95%)** | **P-value** |
| --- | --- | --- | --- | --- |
| LE | Current health expenditure | -0.00 | -0.00 to -0.00 | <0.001 |
|  | Domestic general government health expenditure | -0.00 | -0.00 to -0.00 | <0.001 |
|  | Gross domestic product (GDP) | -0.00 | -0.00 to 3.44e-06 | 0.113 |
|  | Income Index | 18.46 | 16.29 to 20.64 | <0.001 |
|  | Income inequality * | 0.00 | -0.03 to 0.03 | 0.946 |
|  | Years of schooling | -0.05 | -0.19 to 0.08 | 0.433 |
|  | Literacy * | 0.16 | 0.13 to 0.19 | <0.001 |
|  | Education Index | 13.49 | 11.37 to 15.61 | <0.001 |
|  | Unemployment | -0.05 | -0.08 to -0.03 | <0.001 |
|  | Injury’s prevalence | -0.00 | -0.00 to -0.00 | <0.001 |
|  | Intentional homicides * | -0.02 | -0.03 to -0.01 | <0.001 |
|  | Social protection * | 1.14 | 0.82 to 1.45 | <0.001 |
|  | Coverage of social safety net program * | 0.02 | 0.00 to 0.03 | 0.009 |
|  | Urban population (Urbanization) | 0.18 | 0.16 to 0.20 | <0.001 |
|  | Population density | 0.00 | -0.00 to 0.00 | <0.211 |
|  | Poverty * | -0.08 | -0.10 to -0.06 | <0.001 |
|  | PM2·5 air pollution * | 0.02 | -0.00 to 0.04 | 0.102 |
|  | CO2 emissions * | 0.04 | -0.00 to 0.08 | 0.074 |
|  | Air pollution | -0.25 | -0.27 to -0.23 | <0.001 |
|  | Basic drinking-water services | 0.17 | 0.16 to 0.19 | <0.001 |
|  | Managed drinking water services * | 0.02 | 0.01 to 0.04 | <0.001 |
|  | Unsafe water, sanitation, hand washing | -0.16 | -0.18 to -0.14 | <0.001 |
|  | Alcohol consumption | 0.15 | 0.09 to 0.21 | <0.001 |
|  | Prevalence of Alcohol use disorders | -0.00 | -0.00 to 0.00 | 0.066 |
|  | Alcohol use | 0.04 | -0.01 to 0.01 | 0.090 |
|  | Prevalence of Drug use disorders | -0.00 | -0.00 to -0.00 | <0.001 |
|  | Drug use | -7.30 | -9.30 to -5.30 | <0.001 |
|  | Smoking prevalence * | 0.00 | -0.04 to 0.04 | 0.927 |
|  | Tobacco | 0.27 | 0.23 to 0.32 | <0.001 |
|  | Smoking | 0.57 | 0.51 to 0.65 | <0.001 |
|  | Oil consumption * | -0.36 | -0.62 to -0.10 | 0.006 |
|  | Consumption of iodized salt * | 0.00 | -0.02 to 0.03 | 0.658 |
|  | Sugar consumption * | -0.00 | -0.01 to 0.00 | 0.487 |
|  | Prevalence of obesity among adults | -0.61 | -0.67 to -0.55 | <0.001 |
|  | Prevalence of overweight | 0.13 | 0.08 to 0.18 | <0.001 |
|  | HIV & sexually transmitted infections | -0.00 | -0.00 to -0.00 | <0.001 |
| HALE | Current health expenditure | -0.00 | -0.00 to -0.00 | <0.001 |
|  | Domestic general government health expenditure | -0.00 | -0.00 to -0.00 | <0.001 |
|  | Gross domestic product (GDP) | -4.52e-06 | -0.00 to 0.00 | 0.559 |
|  | Income Index | 17.51 | 15.69 to 19.32 | <0.001 |
|  | Income inequality * | 0.02 | -0.00 to 0.05 | 0.062 |
|  | Years of schooling | 0.16 | 0.05 to 0.28 | 0.007 |
|  | Literacy * | 0.11 | 0.09 to 0.14 | <0.001 |
|  | Education Index | 14.64 | 12.79 to 16.49 | <0.001 |
|  | Unemployment | -0.05 | -0.07 to -0.02 | <0.001 |
|  | Injury’s prevalence | -0.00 | -0.00to -0.00 | <0.001 |
|  | Intentional homicides * | -0.03 | -0.05 to -0.02 | <0.001 |
|  | Social protection * | 0.97 | 0.64 to 1.30 | <0.001 |
|  | Coverage of social safety net program * | 0.01 | 0.00 to 0.02 | 0.032 |
|  | Urban population (Urbanization) | 0.15 | 0.13 to 0.16 | <0.001 |
|  | Population density | 0.00 | -0.00 to 0.00 | <0.001 |
|  | Poverty * | -0.07 | -0.09 to -0.05 | <0.001 |
|  | PM2·5 air pollution * | 0.18 | -0.00 to 0.04 | 0.119 |
|  | CO2 emissions * | 0.042 | 0.01 to 0.07 | 0.011 |
|  | Air pollution | -0.21 | -0.23 to -0.20 | <0.001 |
|  | Basic drinking-water services | 0.13 | 0.12 to .015 | <0.001 |
|  | Managed drinking water services * | 0.03 | 0.02 to 0.04 | 0.003 |
|  | Unsafe water, sanitation, hand washing | -0.11 | -0.13 to -0.10 | 0.076 |
|  | Alcohol consumption | 0.12 | 0.07 to 0.017 | <0.001 |
|  | Prevalence of Alcohol use disorders | -0.00 | -0.00 to -0.00 | 0.002 |
|  | Alcohol use | 0.06 | 0.02 to 0.10 | 0.007 |
|  | Prevalence of Drug use disorders | -0.00 | -0.00 to -0.00 | <0.001 |
|  | Drug use | -7.68 | -9.38 to -5.97 | <0.001 |
|  | Smoking prevalence * | 0.00 | -0.3 to 0.03 | 0.868 |
|  | Tobacco | 0.24 | 0.20 to 0.28 | <0.001 |
|  | Smoking | 0.45 | 0.39 to 0.52 | <0.001 |
|  | Oil consumption * | -0.18 | -0.38 to 0.03 | 0.089 |
|  | Consumption of iodized salt * | -0.00 | -0.02 to 0.01 | 0.628 |
|  | Sugar consumption * | 0.00 | -0.01 to 0.01 | 0.775 |
|  | Prevalence of obesity among adults | -0.64 | -0.69 to -0.59 | <0.001 |
|  | Prevalence of overweight | 0.04 | -0.00 to 0.09 | 0.057 |
|  | HIV & sexually transmitted infections | -0.00 | -0.00 to -0.00 | <0.001 |

*** Indicators where the amount of data availability was less than 80%.**

## Supplementary Tables 4 The final list of indicators that entered the econometric model

| **dependent variable** | **independent variables** |
| --- | --- |
| **LE** | Education Index; Unemployment; Injuries prevalence; Urban population (Urbanization); Air pollution; Basic drinking-water services; Alcohol use; Drug use; Smoking; Prevalence of obesity; Sexually transmitted infections |
| **HALE** | Education Index; Injuries prevalence; Urban population (Urbanization); Air pollution; Basic drinking-water services; Alcohol use; Drug use; Smoking; Prevalence of obesity; Sexually transmitted infections |

# Supplementary Figures


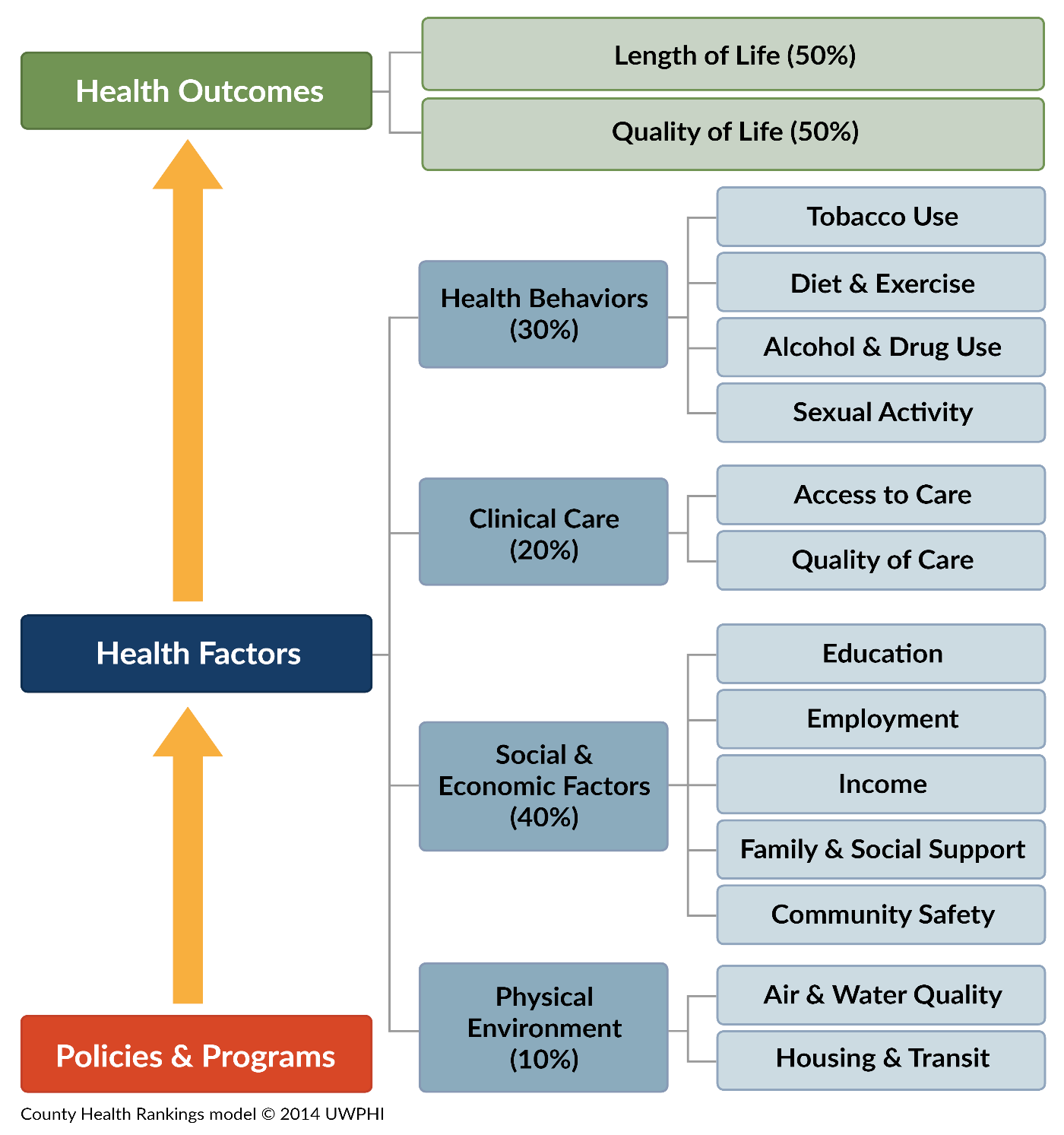


**Supplementary Figure 1.** County Health Rankings & Roadmaps (CHR&R) Model.
